# Retrospective review of clinical and anesthetic findings in 164 heartworm-positive dogs undergoing spay-neuter surgery

**DOI:** 10.1186/s13071-026-07266-8

**Published:** 2026-08-20

**Authors:** Michelle Salob, Brian A. DiGangi

**Affiliations:** University of Florida, Gainesville, FL USA

**Keywords:** Dirofilaria immitis, Spay-neuter, Heartworm, Anesthesia

## Abstract

**Background:**

Timing of spay-neuter surgery in heartworm-positive dogs has been a concern, primarily due to perceived risk of anesthetic complications secondary to cardiovascular compromise. In shelter settings, timing of spay-neuter surgery impacts length of stay and ultimate chance of adoption for heartworm-positive dogs, yet up to 13% of animal shelters may delay spay-neuter procedures until after adulticidal therapy.

**Methods:**

This retrospective review of medical records describes clinical and anesthetic monitoring parameters of heartworm-positive dogs presented for elective spay-neuter surgery in a veterinary student clinical training program. Dogs were assessed for anesthetic/surgical candidacy based on physical examination findings alone and experienced standardized anesthetic protocols.

**Results:**

Patients included 94 females, 69 males, and one intersex patient with ages ranging from 6 months to 15 years. The heartworm status of 73 dogs (44.5%) was unknown at the time of anesthesia and surgery. Noteworthy cardiopulmonary findings on preoperative physical examination included two dogs with a cough and normal heart and lung sounds on auscultation, and five dogs with heart murmurs (grade 2, *n* = 2; grade 3, *n* = 3) with normal lung sounds on auscultation. Two of the dogs with a grade 3 heart murmur were evaluated by a veterinary cardiologist; one was determined to have no pulmonary artery disease, and one was identified as previously diagnosed with mitral valve insufficiency. Average anesthetic time was 98.6 min (range 13–285 min.), 50.3 min for males and 133.7 min for females. Average oxygen saturation (SpO_2_) reading was 96% (range 89–99%); hypoxemia (< 90%) was identified at five specific time points in four patients. Bradycardia (< 60 beats per minutes [bpm]) was identified in four patients (2.4%). There were no perioperative anesthetic emergencies or deaths. A total of nine perioperative surgical complications were identified, six of which were unrelated to heartworm status. The remaining three reports consisted of blood-tinged mucus identified on the endotracheal tube after extubation.

**Conclusions:**

There was no evidence of increased risk of anesthetic or surgical complications in heartworm-positive dogs when screening was limited to a preoperative physical examination and standardized anesthetic protocols were used for elective sterilization procedures.

**Graphical Abstract:**

**Figure:**
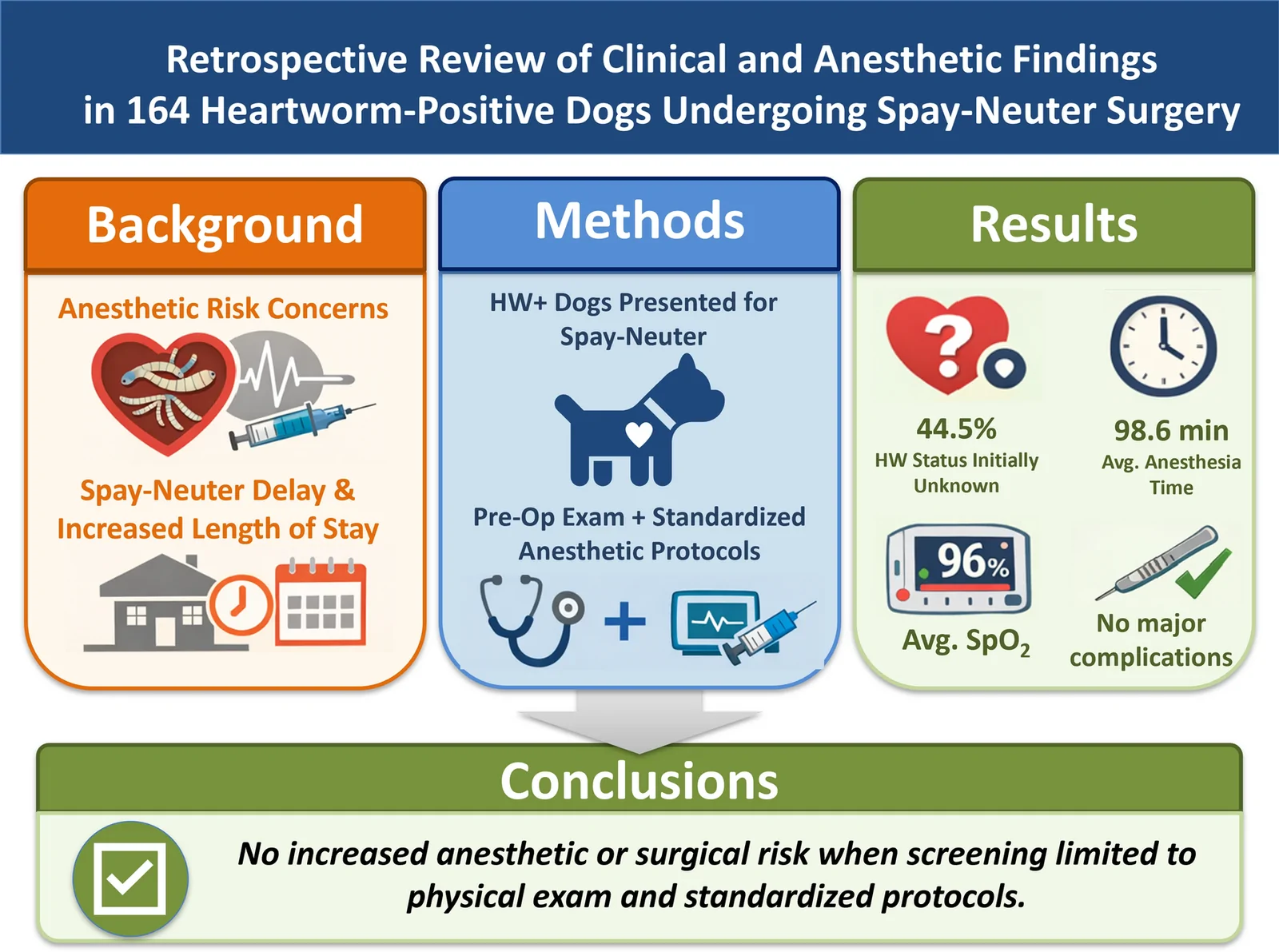

## Background

Positive heartworm status is a common occurrence in shelter dogs across North America and can be a substantial obstacle to timely adoption [1, 2]. At the same time, surgical sterilization prior to adoption is an important practice in animal welfare organizations and, in many jurisdictions, is legally required [3]. Many veterinarians have trepidation about anesthetizing heartworm-positive animals for elective procedures, including as much as 13% of shelters in a 2024 survey reporting delaying spay-neuter surgery until after adulticidal therapy has been completed [4]. However, there is a paucity of literature demonstrating the level of risk, and many veterinarians safely anesthetize and surgically sterilize clinically healthy, heartworm-positive dogs without apparent negative effects.

To date, only one study of clinically healthy, heartworm-positive dogs undergoing spay-neuter surgery has been published [5]. In that report, 15 otherwise healthy (American Society of Anesthesiologists [ASA] status 1 or 2) heartworm-positive dogs were anesthetized using a “cardiovascular-sparing” anesthetic protocol (i.e., avoiding the use of alpha-agonists). These dogs underwent extensive preoperative testing including complete blood count, clinical chemistry profile, heartworm antigen and microfilarial screening, electrocardiogram, and thoracic radiographs, and were monitored for a minimum of 24 h postoperatively. Continuous intraoperative monitoring was also performed by a dedicated anesthetist. Although specific intraoperative clinical parameters were not reported, there were no clinically evident postoperative complications, suggesting that spay-neuter surgery in clinically healthy dogs may not pose undue risks.

Concerns for anesthetic and surgical complications in heartworm-positive dogs stem from sequelae of pulmonary hypertension (e.g., right ventricular dysfunction, right-sided heart failure), pneumonitis and associated inflammation and/or fibrosis, and thrombosis and pulmonary thromboembolism, particularly from dead or dying worms. These processes may result in arrhythmias and/or impaired tissue blood flow and oxygen delivery [6, 7]. Treatment with a combination of pimobendan (inodilator) and sildenafil (pulmonary vasodilator) may improve cardiac output and offset some of these risks [6]. Although advanced diagnostic evaluation to determine the degree of hemodynamic dysfunction present and these pretreatment measures are not accessible in many shelter settings, clinical monitoring for the impact of these changes, particularly in the anesthetized patient, can be relatively noninvasive and straightforward.

The goal of this report is to describe the basic clinical and anesthetic monitoring parameters of heartworm-positive dogs that underwent elective surgical sterilization using standardized clinical, anesthetic, and operational protocols in the absence of adjustments based on heartworm disease status.

## Methods

Hard copies of surgical records of shelter-owned dogs with positive heartworm status presenting for elective spay or neuter surgery at a university-based veterinary student clinical training program between 2011 and 2016 were manually reviewed. Positive heartworm status was based on the documented result of a point-of-care antigen test conducted either postoperatively or previously reported upon clinic admission. Per standard program operating criteria, dogs were eligible for surgery if they were at least 8 weeks of age and were determined to have an ASA status of 1 or 2 based on a preoperative physical examination. Examination included assessment of hemodynamic status (e.g., hydration status, mucous membrane color, capillary refill time) as well as auscultation for cardiopulmonary abnormalities.

Premedication, induction, and maintenance of anesthesia were based on standardized protocols used for adult dogs undergoing ovariohysterectomy and castration procedures. The protocols varied slightly over the 6-year period and consisted of premedication with an opioid (buprenorphine or morphine) and acepromazine, induction with ketamine and a benzodiazepine (midazolam or diazepam) or propofol, and maintenance on isoflurane and oxygen. One patient was anesthetized with a combination of dexmedetomidine, ketamine, and buprenorphine due to temperament. Non-steroidal anti-inflammatory drugs (NSAIDs) were given either by mouth prior to induction (deracoxib or carprofen) or subcutaneously after induction (carprofen). Prior to surgery, testicular blocks for males and incisional blocks for females were performed at the discretion of the surgeon using 2% lidocaine. A splash block was administered to all females following body wall closure using 2% lidocaine. From the time of induction through extubation, patients were monitored by trained technical staff. Automated monitoring equipment was limited to pulse oximeters that continuously recorded hemoglobin saturation and heart rate. Heart rate, respiratory rate, hemoglobin saturation, rectal temperature, and subjective level of anesthetic depth were recorded in the surgical record every 15 min. Surgical procedures were performed by clinical year veterinary students under the direct supervision of experienced spay-neuter surgeons.

Records were examined for descriptors including reported or estimated age, sex, breed, weight, body condition score, and whether heartworm status was determined prior to or after surgery was performed. Physical examination findings and anesthetic monitoring parameters were reviewed and logged individually for each dog. The average of all heart rate, respiratory rate, and hemoglobin saturation values for each individual patient were calculated; individual instances of hemoglobin saturation < 90% were specifically noted. Additionally, records were reviewed for pre-, intra-, or postoperative complications, total surgical time, and total anesthetic time (induction to extubation). All abnormal physical, anesthetic, and perioperative findings were reviewed by the authors for potential relevance to heartworm disease. Summary and descriptive statistics were calculated.

## Results

A total of 164 heartworm-positive dogs underwent elective surgical sterilization in the program over the 6-year period. Patient age ranged from 6 months to 15 years (median = 2.75 years), and the population comprised 94 females (53%), 69 males (42.1%), and one patient with an intersex condition that underwent laparotomy for removal of gonadal tissue (0.61%). Body weight ranged from 2.1 to 38.1 kg (mean = 16.7 kg), and body condition score ranged from 3 to 8 on a nine-point scale (median = 5/9). Ninety-one dogs (55.5%) were known to be heartworm-positive at the time of admission; antigen test results for the remaining 73 dogs were obtained after surgical recovery.

Potentially significant cardiopulmonary findings were described for seven dogs (4.27%). Two dogs (1.2%) had cough only, with normal heart and lung sounds. Five dogs (3.1%) had heart murmurs (three were grade 3/6, two were grade 2/6), all with no history or evidence of coughing and with normal lung sounds. Two of the dogs with grade 3 murmurs received echocardiographic examination by a veterinary cardiologist perioperatively at the client’s request. No evidence of pulmonary artery disease was found in one of these dogs; the other was identified to have had a previous diagnosis of mitral valve insufficiency. No adjustments to the anesthetic or surgical plan were recommended or performed in either of these cases. Abnormal physical examination findings unrelated to heartworm disease included dental disease, ectoparasites, skin disease, injuries, and masses.

Table 1 summarizes the anesthetic monitoring parameters for patients included in this report. There were four dogs with individual instances of recorded hypoxemia (oxygen saturation [SpO_2_] recordings of < 90%). In three of those four cases, only one of the patient’s SpO_2_ data recordings was < 90%, while the rest were normal. One patient had two SpO_2_ data recordings < 90%, while the rest were normal. There were four dogs with individual instances of bradycardia (< 60 beats per minute [bpm]); two of these instances were considered clinically significant and were successfully treated with anticholinergics. There were no other clinically significant anesthetic complications and no perioperative deaths.

**Table 1:** Anesthetic monitoring parameters of 164 heartworm-positive dogs undergoing elective spay-neuter surgery

	Overall (*n* = 157–164)		Females^a^ (*n* = 91–95)		Males (*n* = 66–69)	
	Average	Range	Average	Range	Average	Range
Anesthesia time (min)	98.6	13–285	133.7	35–285	50.3	13–129
Surgical time (min)	70.9	9–233	101.1	21–233	28.4	9–85
SpO_2_ (%)	96	89–99	96	91–99	97	89–99
Heart rate (bpm)	105	70–163	107	72–163	103	65–150
Respiratory rate (rpm)	14	4–51	13	4–25	15	6–51

^a^Includes one intersex patient that underwent laparotomy for gonadectomy

A total of six surgical complications were identified in this dataset. Five of these were instances of intraoperative hemorrhage during ovariohysterectomy, and one was postoperative hemorrhage identified during recovery from orchiectomy. Each of these cases was attributed to surgeon inexperience and was successfully corrected prior to patient discharge.

In three patients (two females and one male), blood-tinged mucous was noted on extubation. These patients recovered uneventfully and had no relevant pre-anesthetic abnormal physical examination findings nor any instances of abnormal anesthetic monitoring recordings.

## Discussion

Elective sterilization of heartworm-positive dogs was performed with no clinically relevant, serious, or disease-related complications when anesthetic candidacy was based on basic physical examination findings alone. Furthermore, no adjustments to standardized anesthetic protocols were made, and in nearly half of the cases reviewed, heartworm disease status was unknown to clinicians at the time of anesthesia and surgery. There were no complications or perioperative deaths noted in the immediate postoperative period. Although the anesthetic and surgical procedures described in this report were performed in accordance with guidelines outlined by the Association of Shelter Veterinarians for spay-neuter programs [8], these data represent a clinical scenario that more closely mimics common practices found in sheltering agencies; high-quality, high-volume spay-neuter (HQHVSN) clinics; and private practice clinics.

Although the outcomes reported here are consistent with those of Peterson et al. [5], there were a number of procedural differences. Pre-anesthetic screening in patients of the current population was largely limited to physical examination findings alone, without the use of complementary diagnostics such as hematological, radiological, and electrocardiological assessments. In the shelter or HQHVSN clinic setting, limiting the preoperative assessment to a physical examination alone is common (especially in the case of young and apparently healthy animals). Although such information might have value, it is likely limited, and there is little justification in resource-limited contexts. In one report of dogs presented to a university-based hospital in Spain, the addition of these complementary diagnostics resulted in an increase in ASA status by 1 level in 3% of patients, and in some cases, findings led to the postponement or cancellation of some elective procedures [9]. Although not specifically tracked, the discovery of heartworm infection alone would have increased the ASA status of many of the dogs in the current population; however, as described, this did not have meaningful impacts on case management or outcomes.

The heartworm disease status of slightly more than half of the current population was determined prior to presentation for spay-neuter surgery. This information was available and could have been known to those performing physical examinations or managing care. While heartworm disease status did not factor into standardized operational protocols, the authors believe the most likely impact would have been in favor of identifying additional abnormalities. That is, those performing physical examinations (typically clinical veterinary students) may have spent additional time auscultating for heart murmurs or assessing hemodynamic status preoperatively. While a heart murmur was detected in five of the known heartworm-positive dogs and none of those without prior heartworm test results, faculty clinicians did not consistently re-auscultate animals examined by qualified students. For these reasons, it is impossible to surmise whether this represents a true bias toward detection of potentially relevant preoperative clinical findings.

The anesthetic and surgical protocols in the current report were not altered to reflect heartworm status, although they largely did not include the use of alpha-agonists. This could have resulted in fewer instances of recorded hemodynamic compromise than if these drugs were regularly included. A recent report described clinical and surgical outcomes in over 1000 dogs with class 1 or 2 heartworm disease (i.e., a population similar to that of the current report) that were anesthetized with the use of an alpha-agonist (dexmedetomidine) as an induction agent. The authors reported 100% survival and only three instances of anesthetic complications including apnea at induction, intraoperative gastrointestinal reflux, and delayed anesthetic recovery [10].

Another differentiating factor between the current population and a typical clinical scenario is the prolonged length of anesthesia and surgical time as compared to typical shelter and HQHVSN practice. Peterson et al. [5] reported mean anesthesia time of 53 min, compared to a mean of 97 min reported here. Given that increasing anesthetic and surgical times are positively correlated with rates of perioperative complications [11, 12], an increase in perioperative complications was anticipated. However, none was appreciated in the data points evaluated, providing additional support for the safety of elective anesthesia and surgery in similar populations of heartworm-positive dogs.

Five instances of intraoperative hemorrhage during spay and one instance of spermatic cord hemorrhage in the immediate postoperative period were documented in the current population. Some spay-neuter surgeons report subjective differences in hemostasis, noting the presence of hyperemic or congested tissue when operating on heartworm-positive dogs. While hypercoagulability, hyperfibrinogenemia, and decreased fibrinolysis have been demonstrated in blood samples from heartworm-infected dogs [13], there were no significant differences in surgical time or intraoperative blood loss between groups of heartworm-positive and heartworm-negative male and female dogs undergoing surgical sterilization in a recent prospective observational study [14]. Although the precise cause of the hemorrhage was not documented for the population reported here, surgeons were inexperienced, and the authors are confident that these instances were most likely due to surgical error (e.g., dropped pedicle, failed ligature), rather than an indication of a clinically relevant manifestation of a disease-related coagulopathy. Similarly, while it is possible that the three instances of muco-hemorrhagic secretions identified on extubation could have been an indication of disease-related pulmonary pathology or coagulopathy, other plausible explanations seem more likely given the absence of any other abnormal findings in these three patients. These include traumatic intubation or endotracheal tube management (e.g., improper sizing, over-inflation of cuff) due to inexperience or an indication of subclinical respiratory disease—a finding that was not uncommon in the clinic population.

Given that this was a retrospective clinical report, there are obvious limitations to the conclusions that may be drawn. Reported parameters are limited to those recorded contemporaneously in the medical records and, while largely objective, cannot be verified, particularly in the case of outlying anesthetic or physical exam data. While physical examinations were mostly conducted by clinical veterinary students and the findings reviewed by faculty clinicians, anesthetic monitoring data were recorded by a variety of staff or volunteers trained in the policies and protocols of the clinic; experience levels, formal training, and academic qualifications of these individuals varied. For these reasons, it is possible that erroneous pulse oximetry readings or patient assessments were not recognized or recorded in the patient records. Although this calls for caution in data reporting and interpretation, this scenario is commonplace in many animal care organizations and offers a realistic perspective on case management.

As described, anesthetic monitoring was limited to manual assessment of physical parameters and the use of a pulse oximeter. While these methods can detect meaningful alterations in hemodynamic status, tissue blood flow, and oxygen delivery, alterations in heart rhythm and cardiac output would be more quickly and accurately assessed with electrocardiography and blood pressure monitoring [6]. Equipment for these purposes was not reliably available where these procedures were conducted, not unlike in many shelters and HQHVSN clinics.

Depth of patient historical information (e.g., administration of heartworm preventives or doxycycline) and length of postoperative follow-up also varied. Most of the dogs in the study had no reported history, as they were recently acquired from local shelters and were presented for care by secondary adoption agencies/rescue groups. All animals were discharged the same day as their surgical procedure, and the onus was on the adoption agency to identify and report complications. Although these partners were traditionally very reliable about reporting concerns, many of these dogs were adopted by community members within days of surgery. Adopters are typically instructed to inform rescue groups in the event of a concern, and it was not uncommon for agencies to maintain ownership of the dogs through the heartworm treatment period. However, the authors did not formally attempt to track outcomes after surgical discharge.

Finally, these data only describe findings in heartworm-positive dogs that underwent surgical sterilization; no comparison was conducted with similarly managed heartworm-negative dogs. It is possible that differences between these two populations exist, and the impact of anesthesia and elective surgery on heartworm-positive dogs might be elucidated by a more direct and/or prospective comparison.

## Conclusions

Current professional guidelines recommend that elective surgical procedures be performed at least 6 months following adulticide treatment with melarsomine [15]. For shelter-housed dogs, this can represent a significant barrier to timely adoption. This is an especially substantial conundrum for shelters where heartworm infection rates have been reported to be as high as 70% [4].

The outcome for shelter-housed dogs with heartworm disease is highly variable, but as many as 34% of shelters do not universally offer heartworm treatment [4]; one report found that over one-quarter (27%) of heartworm-positive dogs in open admission shelters are euthanized due to their disease status [16]. Even when treatment is available, prolonged length of stay, increased financial costs, and negative impacts on quality of life and welfare all impact the larger picture for both the individual dog and shelter operations in general [2].

In a shelter setting, timing of spay-neuter surgery can determine whether or not an animal is eligible for adoption. In heartworm-positive dogs, delay of this surgery may result in significantly increased lengths of stay, which can result in consumption of scarce resources, and may result in euthanasia. The data reported here, in combination with those of the other cited reports, suggest that the perceived risk of anesthetic complications in clinically healthy heartworm-positive dogs may not be of meaningful clinical significance. Based on these findings, the authors support the performance of surgical sterilization in clinically healthy heartworm-positive dogs prior to adulticidal treatment, especially in a shelter setting, where risks of surgical delays and associated costs to the animal and the shelter are far outweighed by the benefits, including a growing evidence base of patient safety.

## Data Availability

Data supporting the main conclusions of this study are included in the manuscript.
